# Erythromelanosis Follicularis Faciei: A Case Report of a Localized Variant

**DOI:** 10.7759/cureus.82029

**Published:** 2025-04-10

**Authors:** Dai Zafer, Renad Althobaiti, Asma S Alabbadi, Khalid Basamih, Wafi Al Hawsawi, Khalid Al Hawsawi

**Affiliations:** 1 General Practice, King Abdulaziz Hospital, Makkah, SAU; 2 Department of Medicine and Surgery, College of Medicine, Umm Al-Qura University, Makkah, SAU; 3 Collage of Medicine, King Saud Bin Abdulaziz University for Health Sciences College of Medicine, Jeddah, SAU; 4 Dermatology, King Abdulaziz Hospital, Makkah, SAU

**Keywords:** erythromelanosis follicularis faciei (eff), facial hyperpigmentation, keratosis pilaris, keratosis pilaris atrophicans, pediatric dermatology

## Abstract

Erythromelanosis follicularis faciei (EFF) is a rare disease, typically seen in late childhood and young adulthood, characterized by symmetrical bilateral hyperpigmented patches and follicular papules on erythematous bases. We present an eight-year-old boy with a three-year history of persistent, asymptomatic erythematous brownish patches and tiny follicular papules localized to the periauricular regions. The lesions worsened in hot climates and were unresponsive to topical steroids and anti-acne treatments. Examination of the hair, nails, and mucous membranes was normal. There was no relevant family history or consanguinity between the parents. The systemic review was unremarkable.

## Introduction

Erythromelanosis follicularis faciei (EFF) is a rare disease that was first described in 1960 by Kitamura et al. [1]. It is common in people of Asian ancestry. The etiology of EFF is still unknown. However, different mechanisms have been suggested, including autosomal recessive inheritance and sporadic mutation [2]. EFF is commonly affecting adolescents or late childhood [3], with a high prevalence in male but female cases have also been described in the literature [4]. The clinical triad of EFF is an erythematous patch, hyperpigmented patch, and follicular papules, EFF begins in the lateral cheeks and preauricular area. When it involves the neck, it is called erythromelanosis follicularis faciei et colli. The bilateral and symmetrical distributions are characteristic of EFF, unilateral cases have been reported [1-3]. In this case, we report an eight-year-old boy with symmetrical EFF that was confined to the preauricular area.

## Case presentation

**Figure 1:**
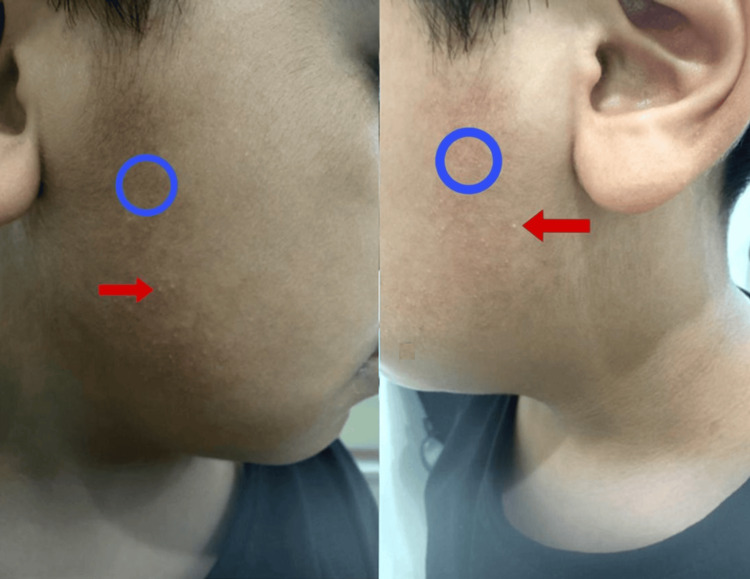
Patient images Non-scaly erythematous–brownish patches and tiny follicular papules over the preauricular area bilaterally and symmetrically. The brownish patches are marked with blue circles, and the follicular papules are indicated by red arrows.

An eight-year-old boy, medically healthy, presented with his parents with a history of asymptomatic persistent skin lesions for three years that were unresponsive to topical steroids and anti-acne treatment. He was started on 0.025% tretinoin cream to be applied every bedtime. He was seen again after three months of treatment with no effect, so Tretinoin was discontinued. Brimonidine 0.33% gel was then initiated and continued for three months, resulting in little benefit. Pulse dye laser was offered to the parents as the next step, but they declined the procedure. The lesions worsened in a hot climate. The review of systems was unremarkable, and there were no similar cases in the family or consanguinity between the parents. Skin examination revealed bilateral non-scaly erythematous-brownish patches and tiny follicular papules over the preauricular areas (Figure 1), with no evidence of scars on the face or scarring alopecia of the eyebrows. Hair, nails, and mucus membranes examinations were normal. The differential diagnosis includes contact dermatitis, post-inflammatory hyperpigmentation (PIH), keratosis pilaris atrophicans, specifically ulerythema ophryogenes (UO) and atrophoderma vermiculatum (AV), capillary malformation, traumatic anserine folliculosis and localized EFF. Parents refused a skin biopsy. Based on the above clinical findings, the diagnosis of EFF was made. 

## Discussion

Our case is unique as there is no previous case of bilateral localized EFF over the preauricular area has been reported in the literature. This rare presentation underscores the need for a comprehensive understanding of the etiopathogenesis of such a condition. Although the patient refused a skin biopsy, this condition is diagnosed clinically. There is a differential diagnosis for such conditions such as PIH, and capillary malformation, contact dermatitis with PIH, keratosis pilaris atrophicans, specifically AV and UO. However, a triad of a patch that has both brownish and erythematous components together with follicular papules are pathognomonic for EFF. Moreover, the parents emphasized that the lesions were there before starting using the topical treatment. They also stressed that the topical treatment neither caused irritation nor accentuation of the lesions. There has been no honeycomb atrophy or atrophic follicular macules that are typical for AV and UO, respectively. UO also commonly involves eyebrows, a feature not found in our patient.

The etiopathogenesis of EFF remains uncertain, with multiple factors proposed as potential contributors. Familial inheritance, particularly an autosomal recessive pattern, has been suggested based on cases of familial clustering. Environmental influences, including temperature fluctuations, ultraviolet (UV) exposure, climate changes, exercise, and emotional stress, are also believed to play a role in both the development and exacerbation of the condition [5,6]. Additionally, autonomic nerve dysfunction has been hypothesized to contribute to the follicular and vascular abnormalities observed in EFF [2]. UV exposure, in particular, has been implicated in triggering and worsening lesions, suggesting a possible photodermatosis-like mechanism. However, the exact interplay between genetic predisposition and environmental triggers remains unclear. Further research, including genetic analysis and long-term studies, is essential to better understand the underlying mechanisms and improve diagnostic and therapeutic approaches [7-9]. While the majority of reported EFF cases present bilaterally, unilateral presentations, though rare, have been documented [10]. This suggests that EFF may encompass a wider clinical spectrum than originally described, necessitating further classification based on lesion distribution and severity [10].

The limitations of the present case report include that it is a single case report. Genetic testing was also not performed which would provide more insight into etiopathogenesis. This study also needed to follow up on the case for a sufficient period to see the effectiveness of the treatment. It also lacks histopathological confirmation to give a more definitive diagnosis. The need for further research on EFF, especially the localized variants, is urgent and important. No treatment has been reported to be effective for EFF. These include keratolytic, topical steroids, corticosteroids, and topical calcineurin inhibitors. We have experienced previous therapies on our patients, but an adequate response has yet to be found effective. However, we started our patient on 0.025% tretinoin cream applied every bedtime and placed him under periodic follow-ups to assess the effectiveness of this treatment. After no improvement, tretinoin was discontinued and brimonidine 0.33% gel was initiated, which also showed little benefit.

## Conclusions

This is a case report illustrating a rare localized EFF in an eight-year-old boy that was localized to the preauricular area. The main differential diagnoses are PIH, AV, and UO. However, the triad of erythema, pigmented patches, and follicular papules are typical for EFF. This necessitates a careful clinical evaluation in patients presenting with unusual skin manifestations. The long-term follow-up is important to see the progression of the disease and its response to various treatments. Further genetic studies would definitely enlighten upon the etiopathogenesis and the clinical spectrum of EFF. Additionally, studies are required to draw more general management guidelines that can be applicable for improving the outcome in a patient population with this condition.
